# A random forest learning assisted “divide and conquer” approach for peptide conformation search

**DOI:** 10.1038/s41598-018-27167-w

**Published:** 2018-06-11

**Authors:** Xin Chen, Bing Yang, Zijing Lin

**Affiliations:** Hefei National Laboratory for Physical Sciences at Microscales & CAS Key Laboratory of Strongly-Coupled Quantum Matter Physics, Department of Physics, University of Science and Technology of China, Hefei, 230026 China

## Abstract

Computational determination of peptide conformations is challenging as it is a problem of finding minima in a high-dimensional space. The “divide and conquer” approach is promising for reliably reducing the search space size. A random forest learning model is proposed here to expand the scope of applicability of the “divide and conquer” approach. A random forest classification algorithm is used to characterize the distributions of the backbone φ-ψ units (“words”). A random forest supervised learning model is developed to analyze the combinations of the φ-ψ units (“grammar”). It is found that amino acid residues may be grouped as equivalent “words”, while the φ-ψ combinations in low-energy peptide conformations follow a distinct “grammar”. The finding of equivalent words empowers the “divide and conquer” method with the flexibility of fragment substitution. The learnt grammar is used to improve the efficiency of the “divide and conquer” method by removing unfavorable φ-ψ combinations without the need of dedicated human effort. The machine learning assisted search method is illustrated by efficiently searching the conformations of GGG/AAA/GGGG/AAAA/GGGGG through assembling the structures of GFG/GFGG. Moreover, the computational cost of the new method is shown to increase rather slowly with the peptide length.

## Introduction

Structures are the basis for understanding the properties and functions of biomolecules such as peptides and proteins. Computational determination of peptide conformations is a challenging problem that searches minima in a high-dimensional space and has remained an active research topic for many years. There are various structural search methods that may be broadly characterized as systematic, stochastic and “divide and conquer”. The systematic structural search method is quite reliable as it considers combinations of all bond rotational degrees of freedom of biomolecule^1^. However, the computational cost of the systematic approach increases exponentially with the size of the molecule and it is applicable to very small peptides^2,3^. The stochastic approach searches the bimolecular structure by sampling its potential energy surface (PES) in some designated way, such as simulated annealing^4–6^, Monte-Carlo^7–9^, genetic algorithm^10–12^, and basin-hopping^13,14^. The stochastic approach is widely used due to its numerical efficiency and almost universal adoptability. However, the reliability of the stochastic approach is often questionable due to the vast search space of the PES. The “divide and conquer” search method first divides a peptide into smaller peptide fragments whose conformations may be reliably determined by, say, a systematic structural search method. The conformations of the constituting peptide fragments are then properly combined to yield the low energy conformations of the target peptide^2,15,16^. The “divide and conquer” method possess a highly desirable feature that the required computational cost increases moderately with the number of amino acid (AA) residues in a peptide^2,17^. Consequently, the “divide and conquer” approach is expected to be useful for the structure prediction of large peptide. In fact, the widely used fragment-based protein structure prediction methods^18–21^ share the spirit of the “divide and conquer” approach, except that the fragment structures in these methods are mined from the Protein Data Bank^22–24^.

When benchmarked with the results of the systematic search method, the “divide and conquer” method has been shown to be both efficient and reliable for determining the structures of small peptides^2,15,16^. However, the existing approach suffers from the following drawbacks: (1) The ensembles of the low energy conformers of the constituting peptide fragments are used to form the trial structures of the target peptide. When the required structural data are not known beforehand, they need to be determined by some systematic searches that can be computationally expensive. The method would be more flexible and more efficient if it only requires the low-energy conformations of related but non-identical peptide fragments that are available in some database. (2) For numerical efficiency, the number of the low energy fragment conformations used for forming the trial structures of the peptide should be minimized. This is made possible by a detailed analysis of the structural features to ensure the chosen fragment structures are capable of forming favorable inter-fragment interactions^2,15,16^. However, the complexity of the analysis increases with the number of AA residues in the peptide. Some approach easily generalizable to larger peptides is desired. (3) The chosen structural sets of the constituting peptide fragments are joined in a combinatorial way. This is inefficient as the dihedral angles (φ and ψ) of the peptide backbone are known to follow some combination rule^3^. In fact, there are strong evidences that the combinations of the fragment structures as reflected by high order φ-ψ plots are highly restrictive^24–28^. The numerical efficiency of the method would be much improved by utilizing the restriction. For example, exploring the high order φ-ψ correlation may result in a dramatic reduction in the size of the peptide conformational space^25,28^.

The goal of this work is to establish a flexible and efficient version of the “divide and conquer” search method by trying to overcome the three limitations mentioned above. First, the low energy conformations of a number of tri- and tetra-peptides are analyzed by the random forest classification^29^ and the multidimensional scaling (MDS) method^30^. The analysis reveals that the peptides may be classified into a few groups with clear similarities in the backbone dihedral angles, indicating the possibility of circumventing the first limitation. Next, a random forest supervised learning algorithm^29^ is used to probe the rule of the φ-ψ combinations in the low-energy peptide conformations. The machine learning approach eliminates the need of a specialized analysis of the fragment structural features so that the second drawback mentioned above no longer exists. The random forest algorithm and the learnt rule of the φ-ψ combinations are then used to screen the trial peptide structures constructed by splicing the fragment conformations. The number of the trial structures is much reduced by the screening and the third limitation is avoided. Applications to representative peptides show that the new method is not only efficient but also highly reliable as demonstrated by comparing with the systematic search results.

## Results and Discussion

### Classification of the φ-ψ units in peptide fragments

**Figure 1:**
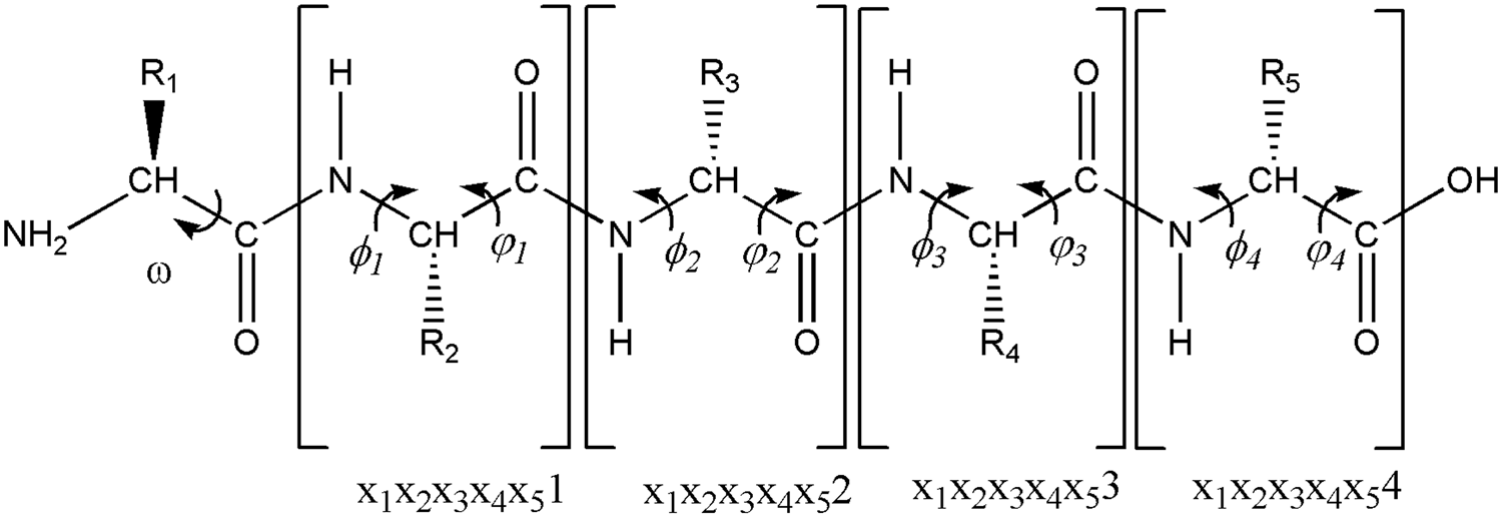
The φ-ψ units in a pentapeptide X_1_X_2_X_3_X_4_X_5_. Generally, x_1_x_2_…x_n_*i* refers to the φ-ψ unit of the (*i* + 1)^th^ AA residue in a peptide X_1_X_2_…X_n_ with *n* AA residues.

A sketch of a peptide with the notations for its backbone dihedral angles is shown in Fig. 1. The φ-ψ units for different AA residues are different by definition. Table 1 shows the “error rate” matrix for different φ-ψ units as predicted by the random forest classification algorithm. The random forest prediction with a high “error rate” for two different φ-ψ units means that the two φ-ψ units are quite similar and not easily distinguishable. Therefore, the error rates shown in Table 1 correspond to the degrees of similarities among different φ-ψ units. As seen in Table 1, the similarity can be quite high for some φ-ψ units, while fairly low for some other φ-ψ units.

**Table 1:** The matrix of “error rate” for different φ-ψ units.

	gvgg1	gvgg2	gvgg3	gtgg1	gtgg2	gtgg3	gfgg1	gfgg2	gfgg3	gtg1	gtg2	gvg1	gvg2	vgg1	vgg2	mgg1	mgg2	fgg1
gvgg2	0.2	—	—	—	—	—	—	—	—	—	—	—	—	—	—	—	—	—
gvgg3	0.2	0.2	—	—	—	—	—	—	—	—	—	—	—	—	—	—	—	—
gtgg1	0.4	0.3	0.2	—	—	—	—	—	—	—	—	—	—	—	—	—	—	—
gtgg2	0.3	0.6	0.2	0.3	—	—	—	—	—	—	—	—	—	—	—	—	—	—
gtgg3	0.2	0.2	0.6	0.2	0.3	—	—	—	—	—	—	—	—	—	—	—	—	—
gfgg1	0.7	0.3	0.2	0.5	0.3	0.2	—	—	—	—	—	—	—	—	—	—	—	—
gfgg2	0.3	0.7	0.2	0.3	0.7	0.2	0.3	—	—	—	—	—	—	—	—	—	—	—
gfgg3	0.2	0.2	0.7	0.2	0.2	0.7	0.2	0.2	—	—	—	—	—	—	—	—	—	—
gtg1	0.4	0.3	0.2	0.5	0.3	0.2	0.4	0.2	0.2	—	—	—	—	—	—	—	—	—
gtg2	0.1	0.1	0.3	0.1	0.2	0.4	0.1	0.1	0.4	0.1	—	—	—	—	—	—	—	—
gvg1	0.4	0.2	0.1	0.3	0.3	0.1	0.4	0.2	0.1	0.4	0.1	—	—	—	—	—	—	—
gvg2	0.1	0.1	0.3	0.1	0.2	0.4	0.1	0.1	0.4	0.1	0.6	0.1	—	—	—	—	—	—
vgg1	0.1	0.3	0.2	0.2	0.4	0.2	0.2	0.4	0.2	0.2	0.2	0.2	0.2	—	—	—	—	—
vgg2	0.1	0.1	0.3	0.1	0.2	0.3	0.1	0.1	0.4	0.1	0.5	0.1	0.6	0.2	—	—	—	—
mgg1	0.1	0.2	0.0	0.1	0.2	0.1	0.1	0.3	0.1	0.1	0.0	0.1	0.1	0.3	0.1	—	—	—
mgg2	0.1	0.1	0.3	0.1	0.1	0.3	0.1	0.1	0.3	0.1	0.4	0.1	0.5	0.1	0.3	0.0	—	—
fgg1	0.1	0.3	0.1	0.2	0.3	0.1	0.2	0.4	0.1	0.2	0.2	0.2	0.2	0.6	0.1	0.4	0.2	—
fgg2	0.1	0.1	0.3	0.1	0.2	0.4	0.1	0.1	0.4	0.1	0.5	0.1	0.6	0.2	0.6	0.1	0.4	0.2

See Fig. 1 for the notions of φ-ψ units.

**Figure 2:**
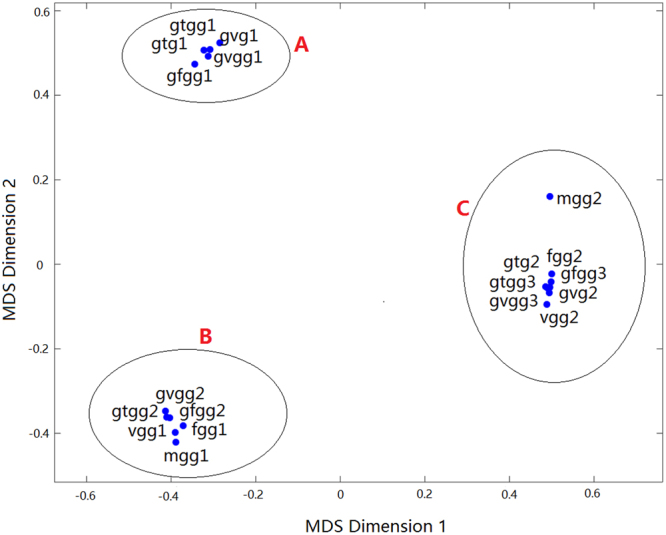
2D MDS map of all φ-ψ units in the low-energy conformations of 8 peptides.

The “error rate” matrix of Table 1 is analyzed by the MDS method. The five largest (normalized) eigenvalues obtained are 1, 0.54, 0.26, 0.22 and 0.16. The two largest eigenvalues are significantly larger than all the other eigenvalues, justifying a dimensional reduction to two-dimension (2D) as a reasonable approximation^30^. The resulting 2D MDS map as an intuitively understandable representation of the φ-ψ units is shown in Fig. 2.

As seen in Fig. 2, all the φ-ψ units can be grouped into three classes: A = (gtg1, gvg1, gfgg1, gtgg1, gvgg1), B = (fgg1, mgg1, vgg1, gfgg2, gtgg2, gvgg2), and C = (fgg2, gtg2, gvg2, mgg2, vgg2, gfgg3, gtgg3, gvgg3). The φ-ψ distributions are similar for a given class, but quite distinct for different classes.

Class A shares a common feature that the AA residues neighboring the φ-ψ unit on both the N- and C-terminus sides are G^31^, even though the AA residue for the φ-ψ unit may be F, T or V. It is generally expected that the φ-ψ distribution should be affected by the AA residue and the neighboring residues of the φ-ψ unit. Class A indicates that F, T and V have similar effects on the φ-ψ distribution. This is possible as no substantial interaction between the side-chain of F/T/V and the peptide backbone is expected. This observation is supported by Class B that shows that F, T, V as well as M have similar effects on the φ-ψ distribution when serving as the N-side neighboring residue. Moreover, Class C further suggests the similarity of G, T and V when serving as the N-side neighboring residue of the φ-ψ unit. However, while the similarity among F, T and V is suggested by both Class A and Class B and partially by Class C, the similarity between G and T/V is only suggested by Class C and should not be over emphasized. In fact, Class A and Class B are quite different due to their difference in the combination of the AA residue and its N-side neighbor of the φ-ψ unit: (F/T/V, G) for Class A and (G, F/T/V) for Class B. It may be said that Class C indicates that G and F/T/V are only mildly different. The clear distinction between Class A and Class B is caused by the amplified effect of the moderate differences in both the AA residue and its N-side neighbor of the φ-ψ unit. The distinction between Class C and Class A/B is quite understandable as the φ-ψ unit in Class C has no C-side neighboring AA residue.

There are tripeptides and tetra-peptides in all the three classes of A, B and C. It is reasonable to conclude that a φ-ψ unit is affected only by its nearest-neighbor AA residues, while the influence of its next nearest-neighbors is negligible. The grouping of the φ-ψ distributions in different peptides and the deduced similarities in AA residues provide the possibility of fragment replacement in the peptide structure construction.

### Testing results on conformational searches

The similarity of the AA residues in their φ-ψ distribution is used to improve the flexibility of the “divide and conquer” conformational search method. The trial structures generated by splicing peptide fragments are screened by the combinational rules of the φ-ψ units deduced from the random forest supervised learning method. The method is applied to the conformational searches of GGG, AAA, GGGG, AAAA and GGGGG. The efficiency and reliability of the method are assessed by comparing with the corresponding systematic search results.

#### GGG and AAA

The initial trial structures for searching the conformations of GGG and AAA are generated by all possible combinations of gfg1 and gfg2 of the low-energy conformations of GFG. By this construction, the similarity between A and F is assumed and the mild difference between G and A/F is believed to be of limited consequence. The trial structures are screened by the random forest model trained by the low-energy conformations of GTG, GVG, VGG, FGG and MGG.

The number of the GGG trial structures surviving the screening is 1,838, as compared to 3,072 generated in a systematic search^2^. Both sets of the trial structures produce the same result for the low energy conformations of GGG, a total of 18 conformers in an energy range of 3 kcal/mol of the global minimum.

The new search method and the systematic search method produce similar but somewhat different results for the conformations of AAA. The systematic search finds 18 AAA conformers that are within 3 kcal/mol of its global minimum, while the new method finds 20 conformers in the same energy range. Combining the results of both the searches and the results of the path matrix method that produced 24 conformers^3^, there are a total of 25 AAA conformers in the energy range. Compared to the systematic search, the new method misses the 18th lowest energy conformers, while produces the new 4th, 11th and 15th lowest energy conformers compared to the systematic method. The path matrix method produces the new 3rd, 6th, 8th and 21st lowest energy conformers. The structures and relative energies of the conformations missed by the systematic search can be found in SI. The missing of conformers is not normally expected for the systematic search method, but has been encountered before^3,12,16^. It may be caused by the geometry optimization process as the relaxation path is not mathematically definite. Alternatively, it may be associated with the semi-empirical method used to optimize the initial structures. Regardless what causes the missing of conformers in the systematic search method, it is observed that the quality of the new method for the AAA conformational search is higher than that of the systematic search method.

#### GGGG and AAAA

The trial structures for the conformational search of GGGG and AAAA are generated by all combinations of gfgg12 and gfgg3 of the low-energy GFGG conformations. The trial structures are screened by the random forest model on the combination of the φ-ψ units trained with the low-energy conformations of GFGG, GVGG and GTGG.

The numbers of the low energy GGGG conformers found within 3 kcal/mol of the global minimum are 19 for both the new search method and the systematic search method. Among the combined results of the two searches, the new method misses the 9th lowest energy conformer, while the systematic search method misses the 15th lowest energy conformer. The quality of the new search results is only slightly inferior to that of the systematic search method. However, the total number of the trial structures used for the conformational search of GGGG in the new search method is 4,069. In comparison, a total of 41,472 or more trial structures were required by the systematic search method^3,16^.

The numbers of the low energy AAAA conformers found to be within 3.5 kcal/mol of the global minimum by the systematic search method and the new search method are 20 and 19, respectively. Among the combined results of 22 conformers, the new method misses the 3rd, 11th and 22nd lowest energy conformers, while the systematic search method misses the 14th and 20th lowest energy conformers (Supplementary Information Fig. S1). The quality of the new search results is moderately inferior to that of the systematic search method, while the number of the required trial structures is reduced by an order of magnitude.

#### GGGGG

The trial structures for the GGGGG conformational search are generated by all combinations of gfgg12 and gfgg23 of the low-energy GFGG conformations. This is a case that the structure of a longer peptide is obtained by splicing the structures of two shorter peptides. Both ggggg2 and ggggg3 thus constructed corresponding to gfgg2, but gfgg2 for ggggg2 and gfgg2 for ggggg3 come from different GFGG conformations in most cases. The 1st and 2nd φ-ψ units of the low-energy conformations of GFGG, GVGG and GTGG are used to train the random forest model. The trained random forest model is used to screen the φ-ψ combinations of ggggg23 in the trial structures of GGGGG. The number of GGGGG trial structures obtained after the screening is 5,438, about two orders of magnitude smaller than the 497,664 trial structures generated by the systematic search method.

The numbers of the GGGGG conformations found within 3 kcal/mol of the global minimum are 13 and 14 for the new search method and the systematic search method, respectively. Only the relatively unimportant 14th lowest energy conformer is missed by the new search method. The quality of the new search is quite satisfactory.

**Figure 3:**
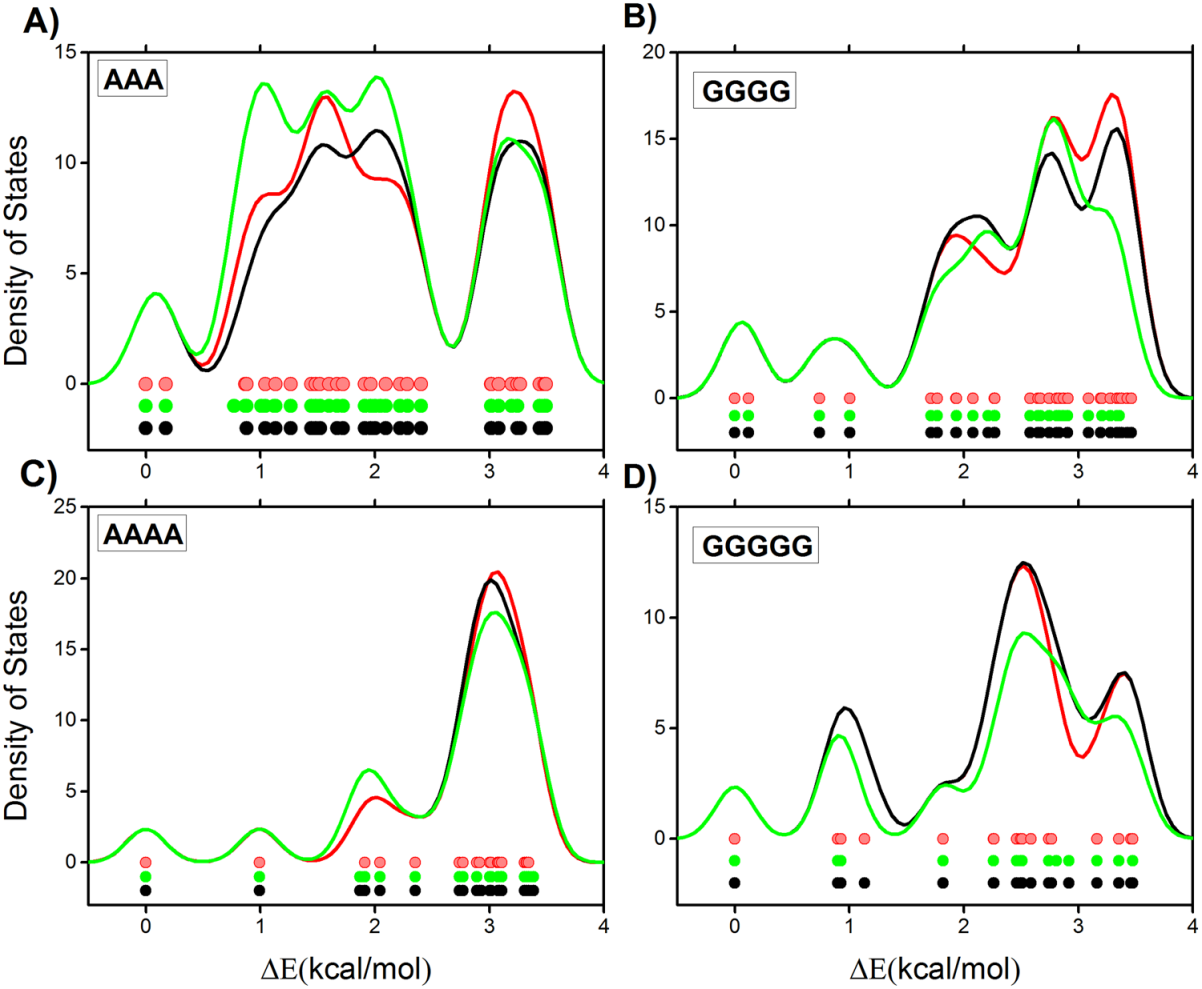
Results of the new search method (in red), the conventional systematic search method (in black) and the path matrix method (in green) on the obtained low-energy conformations of: (**A**) AAA, (**B**) GGGG, (**C**) AAAA, (**D**) GGGGG.

To provide more information about the search results in a succinct way, the energy distributions and the densities of states (DOSs) for the conformations found by the two methods and the path matrix method to be within 3.5 kcal/mol of the global minima are shown in Fig. 3. The DOS contribution of a conformation is represented by a normalized Gaussian, ${\rm{\phi }}({\rm{x}})=\frac{1}{\alpha \sqrt{\pi }}{e}^{-\frac{{(x-E)}^{2}}{{\alpha }^{2}}}$, where the conformational energy, *E*, is relative to the global minimum and α = 0.24 kcal/mol. As can be seen in Fig. 3, the overall quality of the new search results is comparable with that of the computationally intensive systematic searches and the searches by the path matrix method.

### Computational cost of the new search method

As mentioned above, the number of trial peptide structures required by the random forest learning assisted “divide and conquer” method increases slowly with the number of AA residues in the peptide. The number is 1,838 for GGG/AAA, 4,096 for GGGG/AAAA and 5,438 for GGGGG. In comparison, the corresponding numbers for the systematic search method are 3,456, 41,472 and 497,664, respectively^3^. Clearly, the computational efficiency of the new search method relative to the systematic search method is expected to improve dramatically with the increased number of AA residues in the peptide. To be more specific, Table 2 shows the computational costs of the new search method, the systematic search method and the recently proposed path matrix method^3^ for peptides with up to 10 AA residues. The numbers in Table 2 for the systematic search method and the path matrix method are computed analytically^3^. However, the numbers for the new search method are actually determined only for *n* ≤6, while estimated for *n* ≥7 by assuming the number of the low-energy conformations increases by 50 if *n* is increased by 1. Based on the known results for 3 ≤ *n* ≤ 5, the increase of 50 low-energy conformers for an increase of *n* by 1 should be adequate. That is, the numbers shown in Table 2 for *n* ≥7 should be quite realistic.

**Table 2:** Total numbers of trial structures for a peptide backbone with *n* AA residues required by the systematic search method (*N*_sys_), the path matrix method (*N*_PM_)^3^ and the random forest assisted “divide and conquer” method (*N*_RF_).

*n*	3	4	5	6	7	8	9	10
*N*_sys_	3,456	41,472	4.98E + 05	5.97E + 06	7.17E + 07	8.60E + 08	1.03E + 10	1.24E + 11
*N*_PM_	240	1,130	5,310	2.49E + 04	1.17E + 05	5.50E + 05	2.58E + 06	1.21E + 07
*N*_RF_	1,838	4,096	5,438	6,649	7,318	8,540	9,613	11,341

As expected, Table 2 shows that the systematic search method is the most computational intensive for all cases. However, Table 2 shows that the path matrix method is the most efficient for *n* ≤5, while the machine learning based method is the most efficient for *n* ≥6. This is possible because that the computational cost of the former increases by a factor of about 4.7 with the addition of one AA residue, while the increasing factor for the latter is only about 1.2. Consequently, the new search method is recommended when encountering *n* ≥6 and becomes increasingly more favorable with the increase of *n*. Notice that *n* ≈ 10 is known to be the optimal fragment length for the structural assembly in the protein structure prediction^22^. Therefore, the new method should be very useful for improving the fragment based protein structure prediction method by providing reliable structures of peptide fragments in an efficient way. Admittedly, the training set used in this study is limited in size and variety, e.g., lacking the structures of charged residues. The testing result is also preliminary and more studies are necessary. Nevertheless, it is reasonable to expect that the machine learning assisted “divide and conquer” method, with some further improvement, can play a useful role in the structure prediction of peptides and proteins.

## Conclusions

Based on a random forest classification algorithm and MDS analysis, it is found that AA residues can be classified into groups according to similarities in their φ-ψ distributions. A random forest supervised learning model is built to analyze the combinations of the φ-ψ units. It is found that the φ-ψ combinations in truly and not truly low-energy peptide conformations are clearly distinguishable. The two findings are utilized to develop a new “divide and conquer” method for the prediction of peptide conformations. The first finding, the similarity of AA φ-ψ units, increases the flexibility of the “divide and conquer” method by allowing for the peptide fragment substitution. The second finding, the φ-ψ combination rule, improves the efficiency of the “divide and conquer” method by eliminating unfavorable fragment combinations. It also makes the existing “divide and conquer” method more extensible by reducing the need of dedicated human analysis. The new search method is validated by providing excellent results for the conformations of GGG, AAA, GGGG, AAAA and GGGGG. Moreover, a strong advantage of the new search method is that its computational cost increases slowly with the peptide length. Although the testing cases are limited and more studies are required, it is our view that the machine learning assisted “divide and conquer” method can play a useful role in the structure prediction of peptides and proteins.

## Methods

### Systematic search of peptide conformations

Reliable results for the ensembles of the low-energy conformations of representative peptides are needed for both the training and validation of the random forest learning algorithm. The low-energy conformations of the tripeptide set, (GGG, GTG, GVG, VGG, FGG, MGG), the tetrapeptide set, (GGGG, GVGG, GTGG, GFGG), and the pentapeptide GGGGG have been determined by the systematic search method^2,3,16,32^. Here G = glycine, T = threonine, V = valine, F = phenylalanine, and M = methionine. These conformational search results are used here. Notice that the conformations of GGGGG were determined at the level of B97D/6–311++G**/B97D/6–31+G**^3^. For consistency with the results for other peptides presented here, the GGGGG conformations are recomputed at the BHandHLYP/6–311++ G**/BHandHLYP/6–31 G*^33^.

The low-energy conformational ensembles of tripeptides AAA and GFG and tetrapeptide AAAA as well as GGGG are determined here by following the same systematic search procedure (A = alanine). Briefly, initial trial structures of peptides were generated by considering all combinations of their bond rotational degrees of freedom. To lessen the computational burden, the trial structures were first optimized by the semi-empirical PM3 method^34^. The unique structures obtained were sorted by their HF/3–21 G* energies and the low-energy conformers within the range of 20 kcal/mol from their respective global minimum were then optimized at the HF/3–21 G* level. The structures thus determined to be within the 16 kcal/mol range of their global minima were further optimized at the BHandHLYP/6–31 G* level. The single point energies (SPE) for conformers thus found to be within 10 kcal/mol of their global minimum were finally computed at the BHandHLYP/6–311 + + G** level. Unless explicitly specified otherwise, a low-energy conformation in this paper means that its energy is within 10 kcal/mol of the global minimum.

All the geometry optimizations and energy computations were carried out using the GAUSSIAN 09 suite of programs^35^.

### Characterization of the φ-ψ units

The random forest classification algorithm^29^ is used to analyze the φ-ψ distributions in the low-energy conformations of GTG, GVG, VGG, FGG, MGG, GVGG, GTGG and GFGG, referred as the learning set hereafter. The obtained matrix of “error rate” for different φ-ψ units is analyzed by the MDS method. Peptide fragments with the same characteristic φ-ψ distribution are then identified.

### Rule for the combinations of adjacent φ-ψ units

A random forest supervised learning process is employed to learn the pattern of the restricted combinations of neighboring φ-ψ units in the low-energy peptide conformations. A peptide is viewed as consisting of two fragments. The φ-ψ units of a peptide in the learning set belonging to different fragments are allowed to combine with each other in a combinatorial way. A φ-ψ combination is labeled 1 if it is basically the same, by allowing for a noise of 3°, as that found in the low-energy conformations of the peptide. Otherwise, the φ-ψ combination is labeled 0. All the data thus generated are randomly divided into two portions, typically with 60% of the data used as the sample to train and test the random forest classification model. The remaining 40% are used as the independent out of sample test of the learning model. The error rate of the random forest learning model is found to be less than 2% for both in the sample and out of the sample tests. Therefore, the φ-ψ combinations for the low-energy conformations are characteristically distinct from that for other structures. Moreover, tests show that changing the data portion in the sample produces the same results. The learning model is therefore stable for application to the peptide structure prediction.

### Method for the peptide structure prediction

The conformational search of a peptide with *n* AA residues, X_1_X_2_…X_n_ (*n* ≥ 3), starts from the generation of its trial structures. The trial structures are obtained by all combinations of the low-energy conformations of one peptide with *n*_1_ AA residues (the N-side fragment) and another peptide with *n*_2_ AA residues (the C-side fragment). Similar values for *n*_1_ and *n*_2_ are suggested. In this case, the minimal *n*_1_ and *n*_2_ may be found by ${n}_{1}=\,\mathrm{int}(\frac{n\,-\,1}{2})+2$ and ${n}_{2}=\,\mathrm{int}(\frac{n}{2})+1$, respectively. That is, the low-energy conformations of X_1_ × _2_…X_n1_ and X_n1−1_X_n1_ X_n1+1_…X_n_ are combined to form the trial structures of X_1_X_2_…X_n_. After the splicing, the 1^st^ φ-ψ unit of X_n1−1_X_n1_ X_n1+1_…X_n_, x_n1−1_x_n1_ x_n1+1_…x_n_1, is used as the (n_1_ − 1)^th^ φ-ψ unit of X_1_X_2_…X_n_, x_1_x_2_…x_n_(n_1_−1). Notice the notation rule used in this work: capital letters of AA one-letter codes are used when referring a peptide, while small letters are used when referring φ-ψ unit(s) of the peptide. The trial structures are screened by the combination rule for the (n_1_ − 2)^th^ and (n_1_ − 1)^th^ φ-ψ units of X_1_X_2_…X_n1_, x_1_x_2_…x_n_(n_1_ − 2)(n_1_ − 1), as learnt by the random forest supervised learning algorithm from the low-energy conformations of some peptide, …X_n1−2_X_n1−1_ X_n1_…. Notice that larger peptides may generally be used as the fragments for splicing. In such cases, the screening is performed on the combinations of the last used φ-ψ unit of the N-side peptide and the first used φ-ψ unit of the C-side peptide. Only the trial structures surviving the screening are optimized to find the low energy structures of X_1_X_2_…X_n_.

In case that the conformations of a fragment peptide are unknown, they can be substituted by the conformations of a peptide belonging to the same group as the fragment peptide, as learnt by the random forest classification model. Naturally, the side chains are replaced accordingly in the process.

## Electronic supplementary material

Supporting Information
