# Supplementary material for: A random forest learning assisted “divide and conquer” approach for peptide conformation search
Source: Sci Rep. 2018 Jun 11;8:8796. doi: 10.1038/s41598-018-27167-w (PMC5995823; doi:10.1038/s41598-018-27167-w)
Supplement: Supplementary file 1 — Supporting Information [file 41598_2018_27167_MOESM1_ESM.docx]

**A** **random forest learning assisted “divide and conquer” approach for peptide conformation search**

**Xin Chen, Bing Yang, Zijing Lin***

Hefei National Laboratory for Physical Sciences at Microscales & CAS Key Laboratory of Strongly-Coupled Quantum Matter Physics, Department of Physics, University of Science and Technology of China, Hefei 230026, China

^*^Corresponding author. Tel: +86-551-63606345, Fax: +86-551-63606348, E-mail: zjlin@ustc.edu.cn


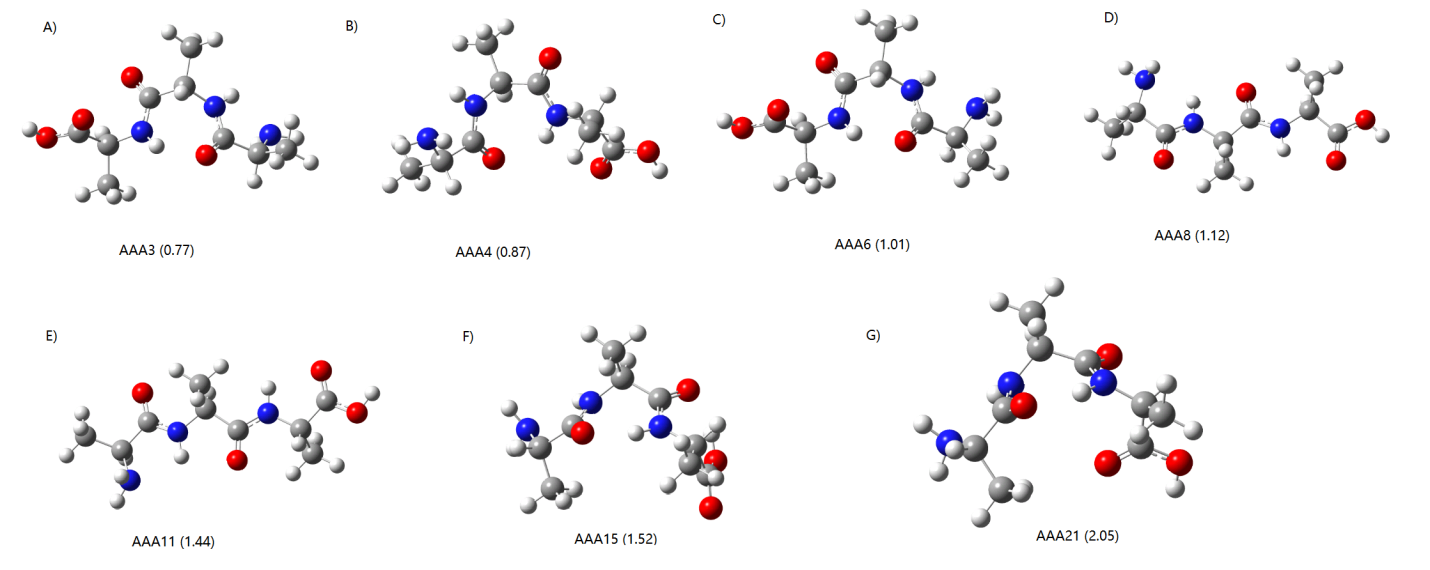


**Figure S1. AAA conformations missed by the systematic search method. Conformational energies (in kcal/mol) relative to the global minimum are shown in the parentheses.**
